# A Comparative study of examination scores and quantitative sensory testing in diagnosis of diabetic polyneuropathy

**DOI:** 10.4103/0973-3930.60007

**Published:** 2010

**Authors:** A. Mythili, K. Dileep Kumar, K. A. V. Subrahmanyam, K. Venkateswarlu, Raju G. Butchi

**Affiliations:** Department of Endocrinology, Andhra Medical College, Visakhapatnam, India; 1 Department of Neurology, Andhra Medical College, Visakhapatnam, India

**Keywords:** 10-g semmes-weinstein monofilament examination, diabetic neuropathy examination score, diabetic polyneuropathy, nerve conduction studies, quantitative sensory testing, vibration perception threshold

## Abstract

**Context:**

Many advances have taken place in the detection of diabetic polyneuropathy with respect to examination scores, electrophysiological techniques and quantitative sensory testing.

**Aim:**

This study aims to evaluate the discriminative power of the Diabetic Neuropathy Examination Score (DNE), 10-g Semmes-Weinstein Monofilament Examination (SWME) and Quantitative Sensory Testing by Vibration Perception Threshold (VPT) in the diagnosis of diabetic polyneuropathy and seek an optimal screening method in diabetic clinic.

**Materials and Methods:**

Hundred consecutive patients with Type 2 diabetes were subjected to Diabetic Neuropathy Symptom Score, DNE score, Semmes-Weinstein monofilament examination, Vibration Perception Threshold and Nerve Conduction Studies; mean ± SD for the various characteristics were calculated. Sensitivity and specificity for the DNE, SWME and VPT were calculated, taking NCS as gold standard.

**Results:**

Seventy one of 100 subjects had evidence of neuropathy confirmed by Nerve Conduction Studies, while 29 did not have neuropathy. The DNE score gave a sensitivity of 83% and a specificity of 79%. The sensitivity of SWME was 98.5% and specificity was 55%. Vibration Perception Thresholds yielded a sensitivity of 86% and a specificity of 76%.

**Conclusions:**

A simple neurological examination score is as good as Vibration Perception threshold in evaluation of polyneuropathy in a diabetic clinic. It may be a better screening tool for diagnosis of diabetic polyneuropathy in view of the cost effectiveness and ease of applicability.

## Introduction

Diabetic Polyneuropathy (DPN) is the most common of the heterogeneous group of diabetic neuropathies and contributes to 50 to 70% of nontraumatic amputations. Screening for diabetic polyneuropathy improves foot care and prevents morbidity. Current level of evidence for optimal screening method is limited. Many advances have taken place in the detection of DPN with respect to examination scores, electrophysiological techniques and quantitative sensory testing. A consensus indicates the need for abnormalities in at least two of five possible modalities to make the diagnosis for research purposes.[1] This study aims to evaluate the discriminative power of the Diabetic Neuropathy Examination Score (DNE), 10-g Semmes-Weinstein Monofilament Examination (SWME) and Quantitative Sensory Testing by Vibration Perception Threshold (VPT) in the diagnosis of diabetic polyneuropathy. The other objective was to seek an optimal screening method in diabetic clinic. The prevalence of various subtypes of neuropathy depending on the distribution was studied.

## Materials and Methods

Subjects included 100 consecutive patients with Type 2 diabetes. Patients were excluded from the study if they had other causes of neuropathy such as alcoholism, liver or renal disease, toxic exposure, other endocrine, metabolic or nutritional disorders, inflammatory diseases, or monoclonal gammopathy. Age, gender, duration of diabetes and history of foot ulceration were recorded. Blood glucose, serum creatinine, routine biochemical and hematological tests, and glycosylated hemoglobin were done in all the subjects. All 100 patients were subjected to:

1. Diabetic Neuropathy Symptom Score
2. Diabetic Neuropathy Examination Score
3. Semmes-Weinstein monofilament examination
4. Vibration Perception Threshold
5. Nerve Conduction Studies

The DNE scores, monofilament test results and vibration perception thresholds were compared against the Nerve Conduction Studies which are taken as a gold standard and the data analyzed.

### Diabetic neuropathy symptom score

All subjects were questioned regarding the presence or otherwise of symptoms, either positive or negative suggesting the presence of neuropathy. The questionnaire was the Diabetic Neuropathy Symptom DNS Score (2) adopted from the Neuropathy Symptom Score (NSS) of Dyck (3).

Diabetic neuropathy symptom Score: The questions should be answered ‘yes’ (positive: 1 point) if a symptom occurred more times a week during the last 2 weeks or ‘no’ (negative: No point) if it did not.

1. Symptoms of unsteadiness in walking?
2. Do you have a burning, aching pain or tenderness of your legs or feet?
3. Do you have pricking sensations at your legs and feet?
4. Do you have places of numbness on your legs or feet?

Maximum score: 4 points; 0 points- PNP absent; 1-4 points - PNP present

(PNP = Polyneuropathy)

### Diabetic neuropathy examination score:

A thorough neurological examination was carried out and the neurological signs were scored following a DNE score, which is a modification of the Neuropathy Disability Score of Dyck.[4] The DNE score consists of eight items, two testing muscle strength, one a tendon reflex, and five sensations. The maximum score is 16. A score of >3 points is considered abnormal.

Muscle strength

1. Quadriceps femoris: Extension of the knee

2. Tibialis Anterior: Dorsiflexion of the foot

Reflex

3. Ankle reflex

Sensation: Index finger

4. Sensitivity to pinpricks

Sensation: Big toe

5. Sensitivity to pinpricks

6. Sensitivity to touch

7. Vibration perception

8. Sensitivity to joint position

Only the right leg and foot are tested.

If right leg is amputated, then left leg is tested.

Scoring from 0 to 2

0 = Normal

1 = Mild/moderate deficit

Muscle strength: MRC scale 3-4

Reflex: Decreased but present

Sensation: Decreased but present

2 = severely disturbed/absent

Muscle strength: MRC scale 0-2

Reflex: Absent

Sensation: Absent

Maximum score: 16 points

A score of > 3 indicates presence of polyneuropathy.

### Semmes-Weinstein monofilament examination

Light touch/pressure perception was assessed using a 10 g monofilament. These were applied on both feet on the plantar surface of the hallux and centrally at the heel. The end of the filament was pressed on the plantar surface of the hallux and centrally at the heel with enough pressure to cause the monofilament to buckle. This was done six times at each point and the participant was blinded to the application of the monofilament during testing.[4] A ‘yes-no’ method was used, meaning that the patient says yes each time he/she senses the application of a monofilament. The ability to correctly sense the monofilament in six trials on both locations was defined as normal, whereas the inability to sense the monofilament correctly in one or more trials was defined as disturbed.

### Vibration perception threshold

VPT was tested using a hand-held biothesiometer (Sensitometer, Dhansai Lab, Mumbai).). After explaining the procedure, the button is applied to various parts of both the feet with the patient relaxed, in the supine position in a quiet room. The vibration is increased gradually from the minimum voltage and the transition from no vibration to the onset of perceiving vibration is taken as VPT. The Yes/No method is used. The VPT is tested on six areas on the plantar aspect of both feet- the hallux, the first metatarsal head, the third metatarsal head, the fifth metatarsal head, the instep and the heel. An average of all the areas tested is taken as the VPT of the subject. The voltage is gradually increased until the patient senses the vibration by the Yes or No. The VPT is measured in volts. In the present study, a voltage of more than 15 V was taken as presence of neuropathy.

### Nerve conduction studies

All patients underwent conventional sensory and motor nerve conduction studies using 2-channel digital electromyograph (Medicaid). The nerves tested were median, ulnar, common peroneal, tibial and sural nerves. The parameters recorded included distal latencies, amplitudes of compound motor action potentials (CMAP), duration of CMAP, F wave latencies and conduction velocities in motor nerves. In sensory nerves, latencies and amplitudes of the sensory nerve action potentials and their conduction velocities were documented.

The presence or absence of neuropathy in these subjects was defined as follows:

**Table:** 

Parameter	No neuropathy	Neuropathy
DNE	3 or less	4 or more
SWME	Normal	Disturbed
VPT	15 volts or less	>15 volts

Means ± SD for the various characteristics were calculated. Sensitivity and specificity for the DNE, SWME and VPT were calculated, taking NCS as gold standard. The relation of diabetic neuropathy to age of the subject, duration of diabetes and glycemic control was determined and their significance was assessed by chi-square test. The Pearson correlation coefficient - r (95% CI) was used to measure the degree of association between the DNE and VPT. A *P* value < 0.05 was considered statistically significant.

## Results

One hundred patients took part in the study and their demographic characteristics are shown in Table 1. Seventy one of 100 subjects had evidence of neuropathy confirmed by nerve conduction studies.

**Table 1:** Demographic characteristics

Number	100
Mean age (years)	52.9 (32-80)
Mean duration of diabetes (years)	6.9 (0-30)
Sex (M/F)	48/52
Mean HbA1c (%)	8
History of foot ulcers (%)	13

**Figure 1:**
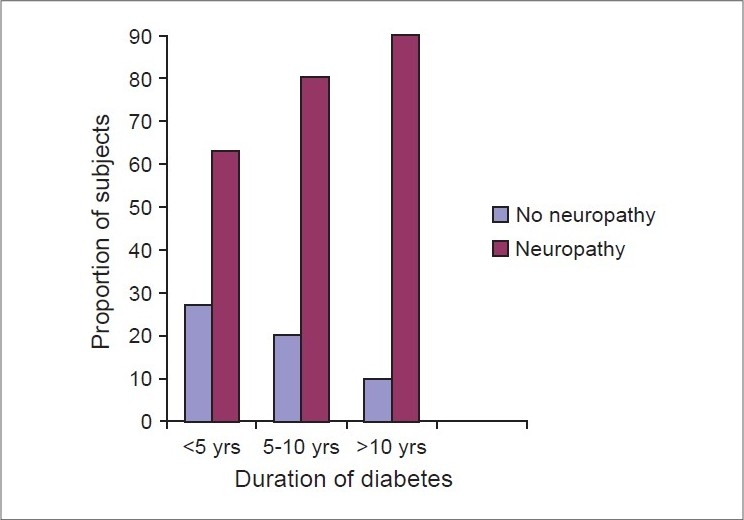
Proportion of subjects with and without neuropathy based on duration of diabetes

The proportion of subjects who had neuropathy with diabetes duration of more than 10 years rose to 90%, compared to individuals less than 10 years (63%). Figure 1 shows proportion of subjects with and without neuropathy based on duration of diabetes.

The mean age, duration of diabetes and HbA1c levels between subjects with and without neuropathy are shown in Table 2.

**Table 2:** Mean age, duration of diabetes and HbA1c in subjects with and without neuropathy

Parameter	No Neuropathy	Neuropathy	*P* value
Mean age	49.34 ± 9.35	54.34 ± 10.21	<0.02
Mean duration of DM(yr)	3.88 ± 4.09	8.09 ± 6.87	<0.01
Mean HbA1c	7.2 ± 1.2	9 ± 1.55	<0.01

The sensitivity and specificity of the DNE Score, SWME, and VPT are shown in Table 3. Out of the 71 subjects who were confirmed to have neuropathy by Nerve Conduction Studies, 59 tested positive by the DNE score which gave a sensitivity of 83%. Of the 29 subjects who were considered as not having neuropathy by the same criteria, 6 had a DNE score positive for neuropathy. The specificity of the DNE score was 79%.

**Table 3:** Sensitivity and specificity of DNE, SWME, VPT

Test	Sensitivity (%)	Specificity (%)	Positive predictive value (%)	Negative predictive value (%)
DNE	83	79	91	34
Monofilament	98.5	55	84	31
VPT	86	76	90	16

Similarly, the SWME was disturbed in 68 of the 71 subjects with neuropathy yielding a sensitivity of 98.5%. SWME was disturbed in 13 of the 29 subjects without neuropathy with a specificity of 55%. Vibration perception thresholds were abnormal in 61 of the 71 subjects with neuropathy, a sensitivity of 86%. The values were abnormal in 7 of the 29 subjects without neuropathy, a specificity of 76%.

**Figure 2:**
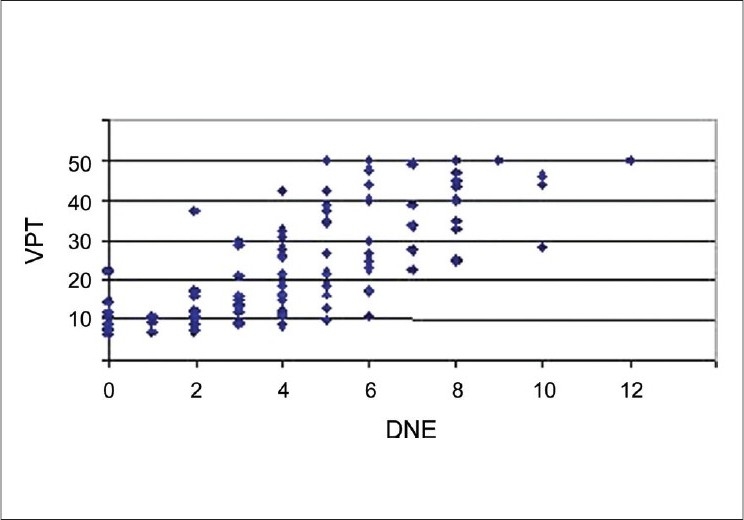
Correlation between diabetic neuropathy examination score and vibration perception threshold

There was a significant correlation between DNE and VPT. Correlation coefficient for the DNE and VPT was 0.72 which was statistically significant. (Pearson's correlation coefficient r = 0.72 with *P* value of < 0.05). Figure 2 illustrates the correlation between DNE and VPT. The various subtypes of neuropathy were analyzed based on nerve conduction studies. Table 4 shows the distribution of various types of neuropathy based on NCS

**Table 4:** Distribution of various types of neuropathy based on NCS

Distal sensory neuropathy	11
Distal sensorimotor neuropathy	54
Distal motor neuropathy	3
Mononeuropathy	2
Asymmetrical	1
Entrapment neuropathies	44

## Discussion

The prevalence of neuropathy in Type 2 diabetes, in the present study, was 71% taking nerve conduction studies as gold standard. Studies on prevalence of neuropathy in Type 2 diabetes had widely differing results, varying from 15 to 50%. The wide variability was attributed to differences in patient sample, diagnostic methods and criteria adopted for diagnosis. In the San Luis Valley Diabetes Study (SLVDS), a population based study of Type 2 diabetic patients where the diagnosis of neuropathy was based on history and examination; there was an overall prevalence of 28%.[5] In another study, the prevalence of DPN in Type 2 diabetes among hospital patients in Spain was 26.7%.[6] However, the study involved assessment of only symptoms and signs for a diagnosis of DPN against a predetermined score. In an Asian hospital-based study, which analyzed the long term complications of newly diagnosed Type 2 diabetes, the prevalence of neuropathy was 25.2%.[7] The higher prevalence of neuropathy in the present study may be due to the selection of patients from the specialty diabetes mellitus clinic who were more symptomatic and tended to have more complications. Another reason for the higher prevalence in the present study could be due to the fact that the diagnosis of neuropathy was established by nerve conduction study which is a more sensitive method. Studies that used nerve conduction studies as a diagnostic marker also reported higher prevalence of neuropathy. A Figure of 45% for prevalence of neuropathy in Type 2 diabetes was reported from a population based sample from Rochester[8] and 42% from a sample of 811 t ype 2 diabetic subjects drawn from 37 UK general practices.[9] In the Rochester study, electrophysiology was used as part of the neurological assessment, but it was a population based study. None of the earlier hospital based studies used electrophysiological studies for diagnosis of DPN.

Duration of diabetes showed a significant effect on the prevalence of neuropathy in the present study. The association between duration of diabetes and the risk of neuropathy is strong and has been confirmed in a variety of studies. In a study from UK, the prevalence of DPN rose from 21% in those with a diabetes duration of less than five years to 37% in people with a duration of over 10 years.[10] In a Spanish study, the prevalence increased from 14% at under five years' duration to 44% at duration of more than 30 years.[6] In the present study, the prevalence was 63% in those with duration less than 5 years to 90% in those with duration more than 10 years. These data probably relate to a bias inherent in a hospital-based study where the more severely affected diabetic patients are taken care of. HbA1c was significantly higher in those with neuropathy in the present study. The risk of developing DPN has been calculated to rise by approximately 10-15% for every 1% rise in HbA1c.[11]

The present study showed a prevalence of 13% for foot involvement, a Figure higher than reported elsewhere. In a population based study from Spain, 3.3% of the subjects had foot ulcers.[6] In an Asian hospital-based study, the ulcer rate was 2.6%.[7] However, this study recruited newly diagnosed diabetes and clinical evidence of neuropathy was seen in only 10% of subjects. The remaining 15% had evidence of neuropathy by Vibration Perception Threshold. The high prevalence of foot ulcers in our study may be due to the higher prevalence of neuropathy. All these ulcers, except in one subject, occurred in patients with neuropathy.

The present study uses the Symptom Score (DNS), and Examination Score (DNE), which were designed by Meijer.[2,4] These scores are simple, reproducible, fast and easy to perform and were modified from the widely used Neuropathy Symptom Score and Neuropathy Disability Score of Dyck. The construct validity of these scores in relation to SWME and VPT were studied earlier.[2,4] The correlation between the DNS and DNE scores and NCS was significant (rho = 0.62 for DNE and 0.51 for DNS).[12] The symptom score was highly sensitive in the present study also, as in the study by Meijer and others. However, since many of the patients included in the study were severely affected they were more likely to have symptoms. Accordingly, the symptom scores were not analyzed further. The sensitivity and specificity of the DNE score was high in this study as compared to nerve conduction studies, sensitivity being 0.83 and specificity of 0.79. This is in concordance with the study by Meijer *et al.*-“Clinical diagnosis of DPN with the DNS and DNE scores”, where both scores are strongly correlated to electro diagnostic studies.[12] The sensitivity and specificity of DNE were 0.96 and 0.51 respectively, for an abnormal result using monofilaments. For an abnormal result using the VPT, these values were 0.97 and 0.59 respectively. Pearson's correlation r for VPT with DNE was 0.75 in Meijer's study[4] and the present study showed similar correlation of 0.72.

A number of cross-sectional studies have assessed the sensitivity of the 10-g monofilament to identify feet at risk of ulceration. Sensitivity varied from 86 to 100%.[13–15] The present study showed sensitivity of 98.5%. The specificity of monofilament in diagnosis of polyneuropathy varied from 45-60% in earlier studies.[16,17] The present study showed a specificity of 55%. Reasons for the poor specificity could be many. The areas chosen for assessment by monofilament varied in different studies. The different areas chosen in various studies were the dorsum of the hallux, ankle, and plantar aspects of great toe, metatarsal heads and heel. In the present study, we utilized the plantar aspect of great toe and heel. Some of the patients had thicker soles, especially the heels where the ability to feel the monofilament was difficult even in the absence of neuropathy. The discrepancies in the use of Monofilament are also with regard to the number of applications, varying from 10 to 1 site. Semmes-Weinstein filaments are available in a number of variable diameters - 1 g, 10 g and 75 g. The 10 g (5.07) monofilament has been shown to be a useful measure of foot ulcer risk in various studies. However, the use of 1 g monofilament in these patients increased the specificity. There are no guidelines as to the application of monofilaments. The filaments that are available also need to be standardized. Booth and Young identified that filaments manufactured by certain companies do not actually buckle at 10 g of force. Indeed, several filaments buckled at <8 g. and could give erroneous results.[18]

Many studies have taken VPT as a gold standard, comparing SWME, and clinical examination with VPT. Dyck and colleagues used computer-assisted QST to compare vibration thresholds with signs and symptoms of neuropathy in three large cohorts: The Rochester Diabetic Neuropathy Study, the recombinant human growth factor study, and the pancreas-renal transplant cohort.[19] In these patient groups, there was a “strong and consistent correlation” between sensory loss and other markers of diabetic neuropathy. Further, the data confirmed that vibration thresholds are especially sensitive to mild or subclinical neuropathy. Davies and co-workers also demonstrated that vibratory thresholds can detect subclinical neuropathy in children and adolescents with Type1 diabetes.[20] Nerve conduction studies subsequently confirmed the neuropathy detected by QST in these young patients. At the other end of the severity spectrum, Boulton and colleagues documented that vibration thresholds provided a strong indication of “risk” for future ulceration across a wide range of ages and durations of diabetes.[21] In the present study, VPT remained highly sensitive (0.86) and specific (0.76) as compared to NCS. Nasseri K and co-workers compared the reproducibility of nerve conduction studies and VPT and concluded that both NCS and VPT are reproducible methods to assess diabetic neuropathy. Determination of VPT has the advantage of being a simple and unobtrusive method.[22]

Analyzing the various subtypes of neuropathy by electro diagnosis, distal sensorimotor neuropathy was found to be the commonest, followed by pure sensory neuropathy. Motor neuropathy was seen rarely, in 3 of the 71 neuropathic subjects. Cranial neuropathy was seen in two cases and asymmetrical axonal in one case. The prevalence of the types of neuropathy in this study is similar to previous data. Carpal Tunnel Syndrome was more common in this series, around 44%.

## Conclusion

The DNE score is quite sensitive in diagnosing DPN. Among the three parameters tested, Vibration Perception Threshold is the most specific. The results of Vibration Perception Threshold are comparable to Nerve Conduction Studies in diagnosing DPN. Monofilament examination, though highly sensitive, was less specific in diagnosing DPN. Duration of diabetes, and HbA1c were positively correlated with neuropathy. Our study showed a higher prevalence of neuropathy and foot involvement compared to the earlier studies. Distal Sensorimotor Neuropathy and Carpal Tunnel Syndrome were the common electrophysiological demonstrable types of neuropathy.
